# Primary angiosarcoma of the oral cavity in a young adult

**DOI:** 10.4322/acr.2020.217

**Published:** 2020-12-08

**Authors:** Bhagat Singh Lali, Zachariah Chowdhury, Monika Gupta, Aseem Mishra

**Affiliations:** 1 Homi Bhabha Cancer Hospital, Department of Pathology, Varanasi, Uttar Pradesh, India; 2 Homi Bhabha Cancer Hospital, Department of Head and Neck Oncology, Varanasi, Uttar Pradesh, India

**Keywords:** Maxilla, Oral Ulcer: Rare Diseases, Sarcoma

## Abstract

Angiosarcoma is a rare neoplasm, constituting only 2% of all the soft tissue tumors and most frequently involves the skin of the head and neck region in elderly males. They are extremely aggressive tumors with high rates of metastasis and poor outcomes. We report a unique case of angiosarcoma involving an unusual site – upper alveolus and maxilla in a young patient highlighting the diagnostic challenges in such a scenario. A 29 years old female presented with a non-healing wound of the oral cavity, which had progressed to the current maximum size of 6.4 cm within one month. Magnetic resonance imaging (MRI) scan revealed the involvement of maxilla up to the floor of the orbit and adjacent soft tissue. However, no distant metastasis was detected on Positron Emission Tomography (PET) scan. Biopsy of the lesion showed an irregular, highly pleomorphic, and mitotically active epithelioid soft tissue tumor conclusively diagnosed as angiosarcoma.

## INTRODUCTION

Angiosarcomas are rare, aggressive malignant sarcomas arising from vascular endothelial cells. They constitute 2% of all the soft tissue sarcomas.^1^ Tumors may arise at any anatomic location, but the skin of the head and neck and breast (arising in chronic lymphedema secondary to modified radical mastectomy) are frequently involved. The typical presentation is a scalp lesion in older Caucasian men.^2^ Angiosarcomas are rare in children and young adults.^3^ They are rapidly progressing tumors that metastasize early and commonly recur locally even after surgical removal. These factors contribute to a reduced overall survival, with almost half of the patients dying within 15 months of diagnosis.^4^ ^,^ ^5^ We present the case of angiosarcoma in a relatively young adult, which appeared at a very rare site, underlining the diagnostic conundrum and pointers towards an accurate diagnosis.

## CASE REPORT

A 29-year-old female presented with a non-healing wound of the oral cavity, which she had had for 1 month. It was rapidly progressing in size. Apparently, she was asymptomatic 1 month earlier, when she developed an ulcer in the left upper alveolus with associated occasional minimal bleeding. She underwent alternative treatment, but the lesion progressed in size. There was no significant family history, and she was a non-smoker, did not drink alcohol, and had no previous history of any chronic illness. On examination, a 4 × 4 cm proliferative lesion in the left upper alveolus was noted, involving the upper gingivobuccal sulcus and the palate (Figures 11B). A blue hue was discerned over the surface of the lesion, and it occasionally bled on touch. There was no cervical lymphadenopathy on palpation.

**Figure 1:**
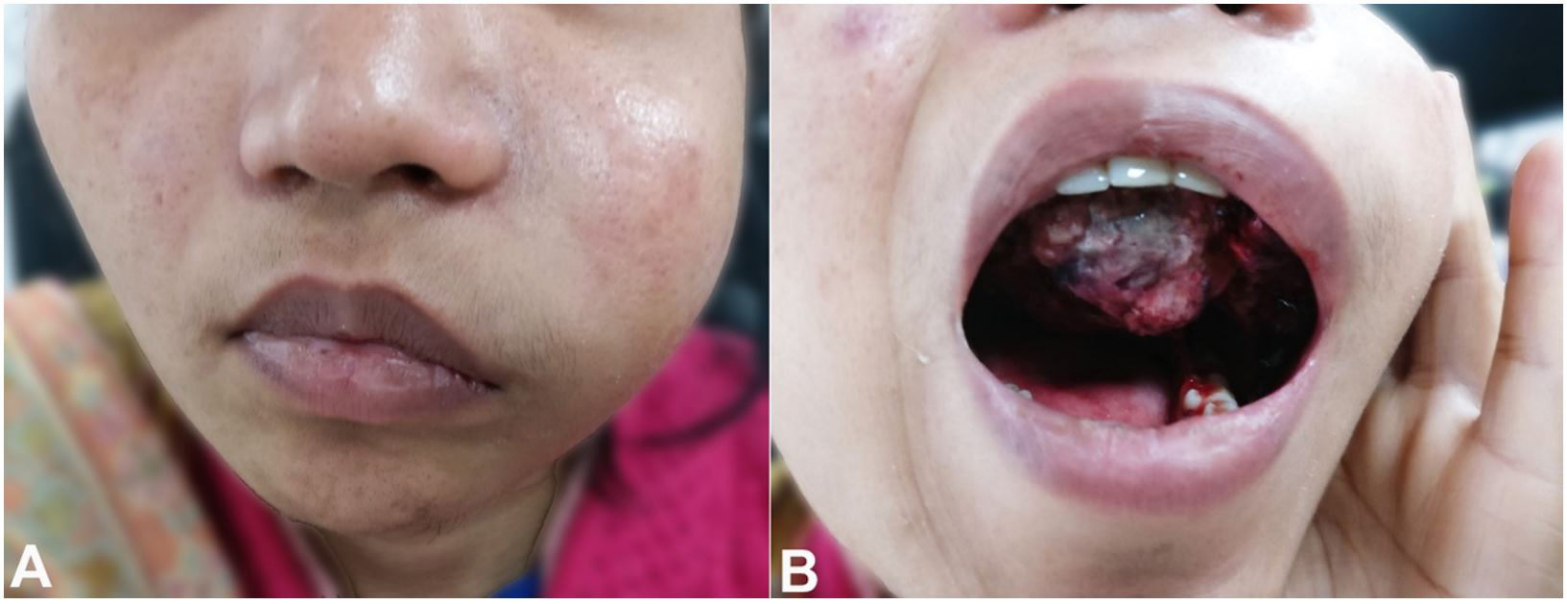
**A** and **B –** Physical examination findings showing marked swelling in the left cheek with upper alveolus and palate involvement.

**Figure 2:**
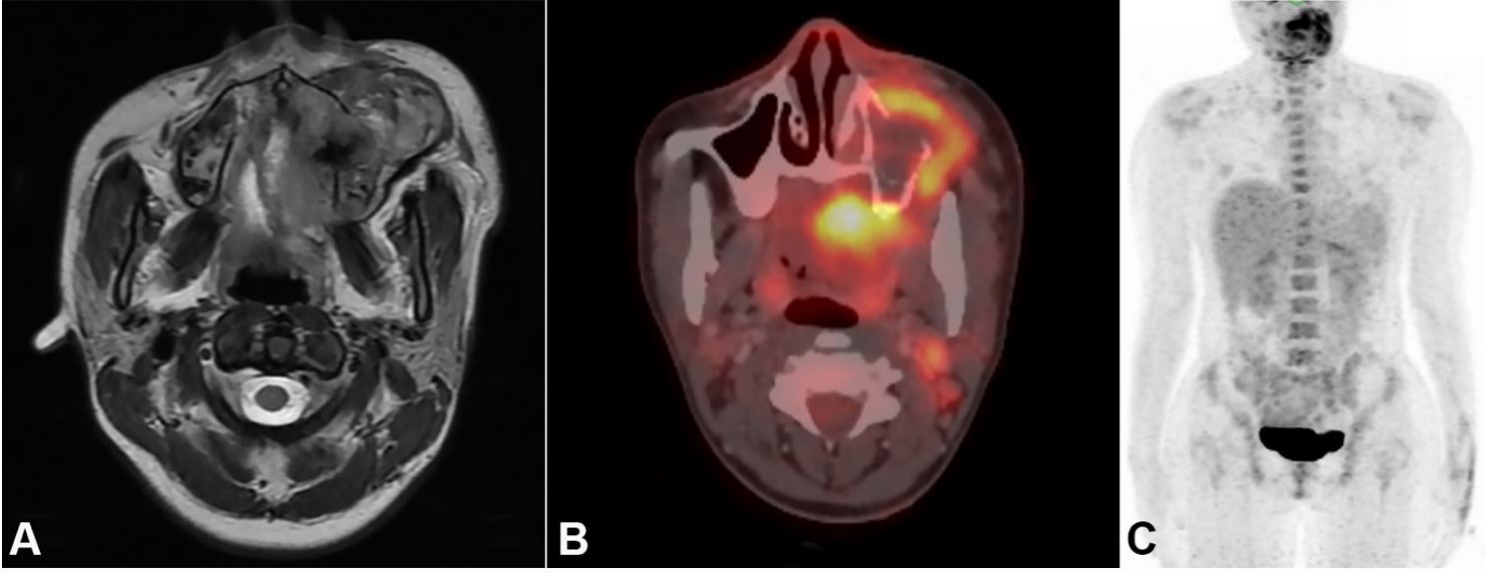
**A –** MRI T2 weighted image displaying the tumor extension; **B –** PET-CT scan demonstrating a fluorodeoxyglucose (FDG) avid tumor; **C –** Whole body positron emission tomography-computed tomography (PET-CT) scan without distant metastases.

A magnetic resonance imaging (MRI) scan of the paranasal sinuses and the neck revealed a heterogeneously enhanced expansile soft tissue mass arising from the left upper alveolus, which measured 6.4 × 5.2 × 5.6 cm (Figure 2A). A computed tomography (CT) scan showed that it had eroded the adjacent alveolar process and lateral wall of the maxilla, extending superiorly into the left maxillary sinus up to the orbital floor, the lateral pterygoid plate up to the skull base, and laterally into the subcutaneous plane. A positron emission tomography-computed tomography (PET-CT) scan revealed a primary avid maxillary tumor (Figure 2B); however, there was no metastatic lesion (Figure 2C).

**Figure 3:**
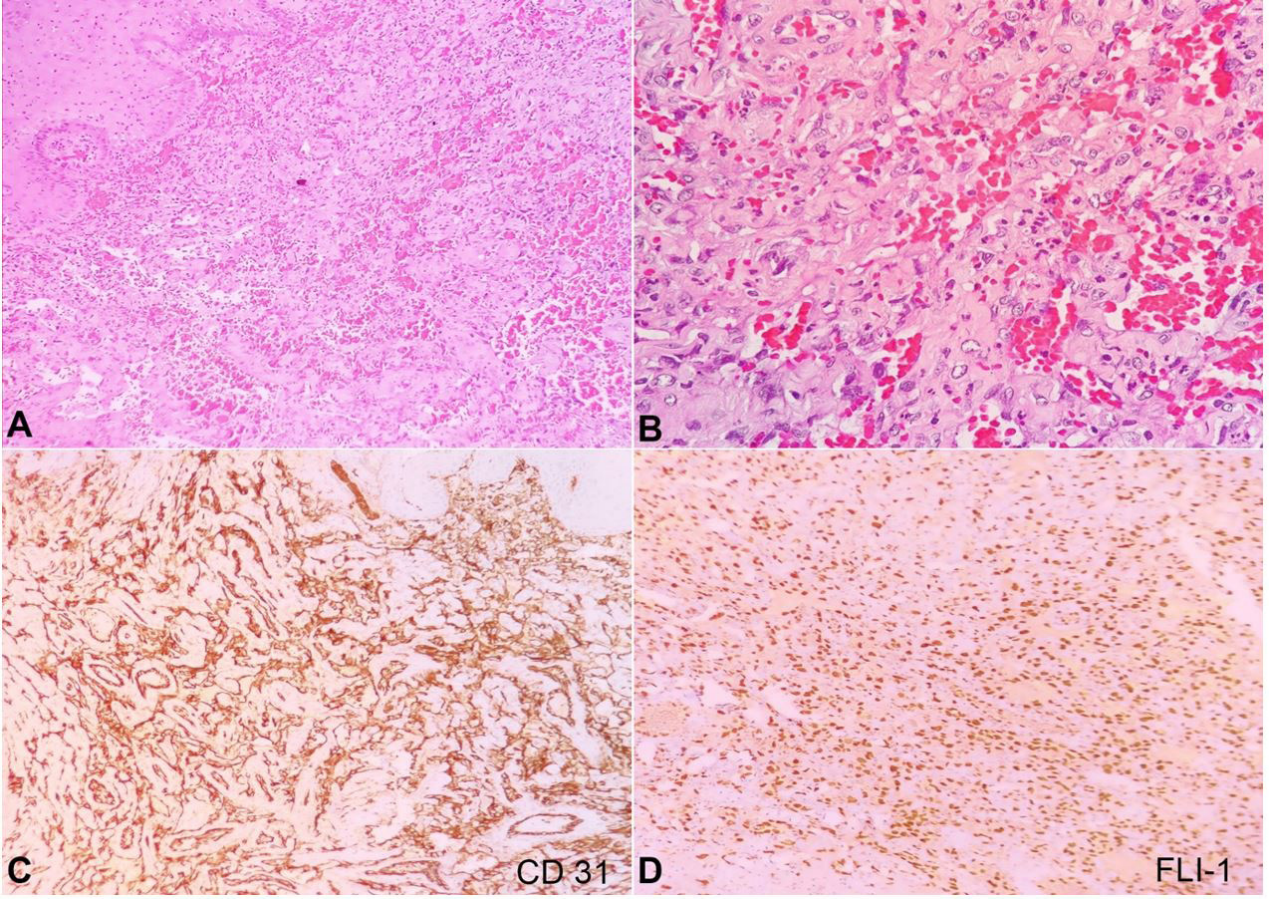
Photomicrographs of the tumor. A – subepithelial irregular vascular tumor (H&E, 10X); B –highly pleomorphic epithelioid tumor cells lining the vascular spaces (H&E, 40X); C – the tumor cells were diffusely positive for CD31 (10X); D – positive reaction to FLI-1 (10X); E – focally positive for CD34 (40X); F – focally positive for AE1/AE3 (10X).

**Figure 4:**
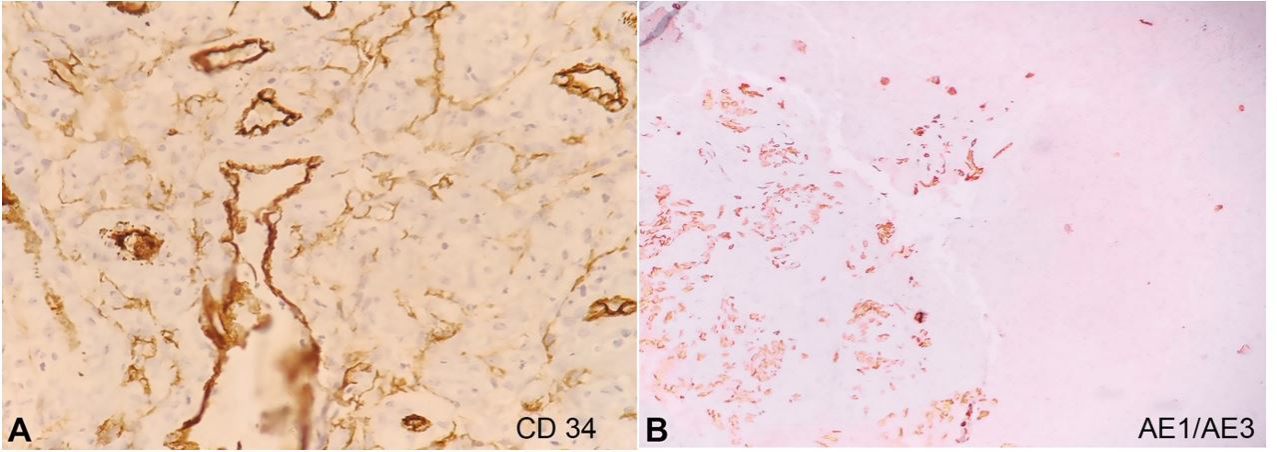
Photomicrographs of the tumor. A- Immunohistochemical reaction focally positive for CD34 (40X); and for AE1/AE3 in B (10X).

A biopsy was performed from the mass; the histopathological examination revealed an irregular subepithelial tumor composed of proliferating vascular channels filled with blood. The lining cells were highly pleomorphic and mostly epithelioid, exhibiting round-to-oval vesicular nuclei along with prominent nucleoli (Figures 33B). Areas of spindle cell proliferation were also identified, as well as large areas of hemorrhage, significant necrosis, and a high mitotic rate (>20/10 hpf). On immunohistochemistry (IHC), the tumor cells were positive for CD31 (Figure 3C), FLI-1 (Figure 3D), CD34 (Figure 4A) and focally for AE1/AE3 (Figure 4B). The Ki-67 proliferation index was approximately 75-80% in the highest proliferation zone. These features pointed towards a diagnosis of angiosarcoma.

Due to the involvement of the lateral pterygoid plate and the skull base, the tumor was inoperable. The patient was scheduled for palliative chemotherapy and radiation, but she did not turn up for the treatment.

## DISCUSSION

Angiosarcoma is one of the rarest soft tissue tumors. It occurs most commonly in the skin or subcutaneous tissue of sun-exposed areas due to ultraviolet light exposure.^1^ ^,^ ^2^ Most of the cases are sporadic, and the potential causes of occurrence include radiation exposure, chemicals, or chronic lymphedema.^4^ History of trauma is cited as one of the causes, but authors believe it merely draws attention to a pre-existing lesion.^1^ However, our patient did not have any history of exposure to these risk factors.

Angiosarcoma is seldom encountered in young individuals, and our patient was only 29 years old. The usual mean age of patients is more than 70 years. In the SEER database of 1,250 primary head and neck angiosarcoma, only 1.3% of patients were younger than 30 years.^2^ Angiosarcoma is associated with congenital diseases, such as neurofibromatosis, bilateral retinoblastoma (Rb1 deletion), Klippel-Trenaunay syndrome, Aicardi syndrome, or xeroderma pigmentosum, which suggests a genetic association in young patients.^6^

Males are more commonly affected than females (2-3:1). Additionally, the site of involvement in our patient was very rare. In the SEER database, 93.5% of tumors were found to be cutaneous, and only 1.6% (n = 20) involved facial bones, buccal mucosa, or gums.^1^ ^,^ ^2^ Occasionally, metastatic tumors to the oral cavity have been reported from a primary tumor in the scalp or other locations.^7^

The clinical differential diagnosis in the present case included squamous cell carcinoma, hemangioma/vascular lesion, melanoma, or metastatic disease. Squamous cell carcinoma was included because it is the most common oral malignancy;^8^ vascular tumors due to the bluish hue over the lesion; and melanoma or metastatic disease due to the rapid growth of the lesion.

Rendering a diagnosis of angiosarcoma in an oral cavity lesion in a relatively young adult patient is a difficult proposition, due in part to (i) the rarity of the entity in this age group; (ii) the uncommon site; and (iii) the significant clinical consequences of this diagnosis. The histomorphology can be variable, ranging from highly differentiated tumors resembling benign or intermediate-grade vascular lesions (e.g., hemangioma or hemangioendothelioma), to anaplastic lesions that are difficult to distinguish from a poorly differentiated carcinoma or other high-grade sarcomas.^9^

The typical picture is composed of anastomosing vascular spaces lined by atypical plump epithelioid-to-spindle cells with multilayering, and brisk mitosis and necrosis. In contrast to hemangiomas, the vascular channels in angiosarcoma seem to create their tissue planes, dissecting through the collagen and fascia; the tumor cells usually have larger hyperchromatic nuclei and often pile up along the lumens creating papillations.^6^ Hemangioendotheliomas can be excluded in the presence of extensive necrosis, marked cytologic atypia, epithelioid morphology, and brisk mitosis.^3^ An epithelioid morphology, a lack of obvious vasoformative foci, and frequent cytokeratin positivity may lead to an erroneous diagnosis of poorly differentiated metastatic (or primary) carcinoma.^6^ Pseudovascular adenoid or acantholytic squamous cell carcinoma can mimic angiosarcoma in the oral cavity on histology.^10^ Histopathological features, which are helpful in distinguishing epithelioid angiosarcoma from poorly differentiated metastatic/primary carcinoma, include occasional intracytoplasmic vacuoles with or without red blood cells, anastomosing vascular channels, and the lack of a desmoplastic reaction encountered in the former. Furthermore, strong immunoreactivity for vimentin, coupled with endothelial cell markers (Factor VIII, CD31, CD34) positivity, renders the diagnosis of metastatic carcinoma unlikely. The other differential diagnostic possibilities include malignant melanoma and epithelioid sarcoma.^9^ ^,^ ^11^ In addition to subtle histological differences, a panel of IHC markers, including positive staining for vascular markers and negative immunostaining for HMB-45, Melan-A, S 100, and CD45 readily exclude the diagnostic mimics.^11^ ^,^ ^12^ Notably, INI-1 was retained in our case, which helped to rule out an important mimicker—namely epithelioid sarcoma.^13^

Immunostaining with vimentin shows strong and diffuse positivity in angiosarcoma. The endothelial markers, CD31 and Factor VIII, are positive in nearly 100% of cases. CD34 positivity ranges from 40% to 100%;^12^ however, it is not a specific marker and is positive in very close morphological mimics, namely epithelioid sarcoma and solitary fibrous tumor. FLI1, a nuclear transcription factor, identifies more than 95% of vascular tumors regardless of the type and grade.^14^ However, it is also expressed in Ewing sarcoma, subsets of a wide range of mesenchymal tumors, subsets of high-grade lymphomas including lymphoblastic lymphomas and diffuse large B-cell lymphomas, and even subsets of carcinomas and melanomas. Thus, it has limited utility by itself owing to its low specificity.^11^ Another nuclear transcription factor, ERG, is an exquisitely sensitive endothelial marker of vascular differentiation and is retained in even poorly differentiated angiosarcomas.^13^ However, ERG is also expressed in a subset of Ewing sarcomas (5-10%), prostatic adenocarcinomas, and a small subset of acute myeloid leukemia.^11^ Among the vascular markers, CD31 is the most sensitive and specific antigen for endothelial differentiation; in combination with FLI-1, it helps the confident diagnosis of angiosarcomas.^1^ ^,^ ^6^ ^,^ ^9^ CD31 and FLI-1 positivity can be misleading, and a mistaken diagnosis of atypical fibroxanthoma is sometimes made; however, morphological details can help to avoid this.^1^ Cytokeratin and epithelial membrane antigen positivity has been reported in up to 50% of epithelioid angiosarcomas focally, which was our finding as well.^15^ These markers could be potential diagnostic pitfalls and quandaries, so they should be used and interpreted in the appropriate clinicopathologic settings.

Surgery is the modality of choice for squamous cell carcinomas or sarcomas. Since the tumor was inoperable in our case due to the lateral pterygoid plate’s involvement up to the skull base, palliative chemotherapy and radiation were the treatment options available. The definite histopathological diagnosis in such cases has major treatment implications. Squamous cell carcinomas are radiosensitive while sarcomas or melanomas do not respond to radiotherapy.^16^ Advanced malignant melanomas are tested for BRAF V600E mutations, and based on that, they are treated either with BRAF inhibitors and MEK inhibitors, or immunotherapy.^17^ In the case of metastatic carcinoma, the therapy is based on the primary tumor type. On the other hand, paclitaxel-based systemic chemotherapy is the preferred regimen for angiosarcomas. Tyrosine kinase inhibitors (TKI) have been used to inhibit the vascular endothelial growth factor (VEGF) signaling pathway. Although a few studies have reported some promising results with TKI (e.g., sorafenib), there is no proven benefit.^18^ The other treatment options include chemotherapy with recombinant interleukin-2, or radiotherapy in a few cases, especially in the form of concurrent chemoradiation.^19^

However, the prognosis in angiosarcomas is dismal owing to early metastasis and multiple lesions. Up to 32% of patients have metastatic lesions at the time of diagnosis.^20^ The 5-year overall survival and disease-free survival is 26.5% and 48.3%, respectively.^2^ In multivariate analysis, age ≥70 years, tumor size of ≥5 cm, and the presence of metastasis at the time of diagnosis were the independent prognostic factors. Among them, the presence of metastasis is the most important since its presence increases the risk of overall and disease-specific death by 197% and 399%, respectively.^2^

To conclude, we present an exceedingly rare angiosarcoma case with respect to the unusual age at presentation and the site of involvement. Within the oral cavity, the differential diagnoses are varied and may be challenging in various scenarios. The pathology pearls must be borne in mind to surmount the diagnostic challenges. The treatment modalities also need to be tailored, considering the whole scenario. Nevertheless, the overall survival of patients is grim with these aggressive sarcomas.
